# Association between postoperative radiotherapy for young-onset head and neck cancer and long-term risk of second primary malignancy: a population-based study

**DOI:** 10.1186/s12967-022-03544-y

**Published:** 2022-09-05

**Authors:** Xiaoke Zhu, Jian Zhou, Liang Zhou, Ming Zhang, Chunli Gao, Lei Tao

**Affiliations:** Department of Otolaryngology, Shanghai Key Clinical Disciplines of Otorhinolaryngology, Eye Ear Nose & Throat Hospital, Fudan University, 83 Fenyang Road, Shanghai, 200031 People’s Republic of China

**Keywords:** Head and neck squamous cell carcinoma, Second primary malignancy, Postoperative radiotherapy, SEER, United States

## Abstract

**Background:**

Second primary malignancy (SPM) represents the leading long-term cause of death among patients with index head and neck squamous cell carcinoma (HNSCC). We aimed to quantify the association between postoperative radiotherapy (PORT) and the risk of SPM development for index HNSCC among adolescent and young patients, who are particularly vulnerable to radiation-associated impacts due to their increased tissue susceptibilities and longer life expectancies.

**Methods:**

This study was conducted using the Surveillance, Epidemiology, and End Results (SEER) database to collect the data of 5 year survivors of index young-onset HNSCC from 1975 to 2011. The outcome of interest was SPM, a new, metachronous malignancy after the index HNSCC. Standardized incidence ratios (SIRs) and excess absolute risks (EARs) were used to quantify the PORT-associated risks externally, and relative risks (RRs) were estimated by the multivariate Poisson regression analysis to quantify the PORT-associated risks internally.

**Results:**

Of the included 2771 5 year survivors with index young-onset HNSCCs, the receipt of PORT (37.6%) was associated with higher risk of SPMs (RR, 1.23; 95% CI 1.07 to 1.43). PORT-associated risks were elevated for the majority of sites, including head and neck (RR, 1.19; 95% CI 0.95 to 1.50) and lung (RR, 1.67; 95% CI 1.18 to 2.34). With regarding to the subsites of head and neck, RRs were above unity in oral cavity squamous cell carcinoma (SCC) (RR, 1.68; 95% CI 1.39 to 2.03) and laryngeal SCC (RR, 1.02; 95% CI 0.73 to 1.43). A relatively greater RR was observed for patients younger than 35 years (RR, 1.44, 95% CI 0.37 to 5.57) and those diagnosed with localized diseases (RR, 1.16, 95% CI 0.9 to 1.5). PORT-associated risks were increased remarkably after 15 years of follow-up (RR, 1.24; 95% CI 0.97 to 1.58).

**Conclusions:**

An association was discovered between PORT treatment and increased long-term risk of SPM among patients with index young-onset HNSCC. The findings suggest long-term follow-up surveillance for these patients, particularly those with oral cavity SCC or laryngeal SCC.

**Supplementary Information:**

The online version contains supplementary material available at 10.1186/s12967-022-03544-y.

## Introduction

Head and neck cancer (HNC) is the seventh most common cancer worldwide. It involves all malignancies originating from the anatomic subsites of the upper aerodigestive tract, including the oral cavity, larynx, oropharynx and hypopharynx [1]. Globally, there were 4,100,000 prevalent cases and 1,100,000 new cases of HNC in 2016, leading to approximately 500,000 deaths [2]. Of these malignancies, SCC constitutes 90% of histological types in HNC [3]. The median age of patients is approximately 60 years, yet, an increasing trend of young-onset HNSCC in people younger than 45 years was reported worldwide. This may be due to the increased incidence of young-onset tumors affecting the oral cavity and oropharynx [4–6].

The cancer treatment guidelines are not significantly differentiated between young and older patients. Surgery remains the mainstay of treatment for young-onset HNSCC, emphasizing performing en-bloc resection with a clear pathological margin greater than 5 mm, and achieving a good aesthetic and functional outcome and quality of life [7, 8]. After surgical resection, adjuvant radiotherapy with standard dose and volume recommended by guidelines [9] serves as a critical supplementary treatment for subclinical foci elimination. It is reserved for patients with adverse features, such as advanced T stage, multiple positive lymph nodes, lymphovascular or perineural space invasion, and positive margins [10]. However, it has been reported in many cancers that PORT could result in elevated risks of long-term adverse consequences, including SPM [11–13].

It should be noted that SPM represents the leading long-term cause of death among HNSCC patients, attributing to approximately 30% of overall deaths, triple the number of mortalities resulting from distant metastases [14, 15]. Only a few studies have explored the association between radiotherapy and SPM incidence in HNSCC. Nevertheless, the results were equivocal. Some studies have demonstrated an increased risk of field cancerization due to radiotherapy [16], but others suggested that radiotherapy could reduce the incidence of SPM in HNSCC [17, 18]. Thus, no conclusive evidence has been found on the association between radiotherapy and SPM incidence in HNSCC. This may be caused by the following limitations: short follow-up, small sample size, or failure to consider a 5-year minimum latency span between radiation exposure and SPM development.

The adolescent and young population are particularly vulnerable to radiation-associated impacts due to their increased tissue susceptibilities and longer life expectancies [19, 20]. Therefore, the impact of postoperative radiation on SPM development requires further investigation among these patients. This study aimed to examine the risk of SPM related to postoperative radiation for young-onset HNSCC using the US SEER-9 database and incorporate approximately more than 15 years of the follow-up period.

## Methods

### Study population

Patients diagnosed with a first primary young-onset HNSCC (oral cavity, larynx, oropharynx, hypopharynx) between 1975 and 2011 within nine SEER registries were eligible, representing a wide cross-section of the United States population with regards to ethnicity, education, and income level [21] (Additional file 1: Table S1). We have selected 45 years as the cutoff point for consistency with previous studies and the American Joint Committee on Cancer (AJCC) staging definitions [22, 23]. Only patients with an accurate record of the sequence of surgery and radiotherapy were included in this study. Patients receiving radiotherapy alone and those receiving intraoperative radiation or radiation prior to surgery were excluded. Postoperative radiotherapy was defined as receiving “beam radiation” after surgery. According to the SEER Combined summary stage and Historic Stage A variables, cases with distant metastasis at diagnosis were also excluded. Sensitivity analysis was conducted by restricting young-onset HNSCC cases to those younger than 40 years at diagnosis.

### Outcome definition

The outcome of interest was SPM (Additional file 1: Table S2). SPM was defined as a new, metachronous invasive malignancy after the diagnosis of index HNSCC, under the criteria proposed by Warren and Gates [24] and further modified by the National Cancer Institute [14, 25]. The second cancer was recorded as an SPM if it developed in a different location, or it was of non-squamous cell origin. If the second cancer was of squamous cell origin and located in the same region as the primary HNSCC, it was also considered as an SPM when diagnosed 60 months after the index diagnosis. Considering the complexity of SPM diagnosis and the high likelihood of incident malignancy detection immediately after HSNCC diagnosis, follow-up for the analysis of SPM (consistent with clinical opinion) was initiated 60 months after the diagnosis of early-onset HNSCC, taking the minimum latency for radiation-induced cancerization into account [26]. The endpoint for follow-up was SPM diagnosis, death or last follow-up time, whichever happened the earliest.

### Statistical analysis

The analyses consist of two objectives. For objective 1, consistent with previous SEER-based studies, we externally compared the risks of SPM for PORT-treated and non-PORT-treated patients with that of the general population, respectively, by estimating SIRs and EARs. SIR is defined as the ratio of observed (O) to expected (E) number of malignancies (SIR = O/E), in which the expected number is derived from a reference SEER population of an identical calendar year, age, race, and sex. EAR represents the absolute number of excess subsequent malignancies attributed to index early-onset HNSCCs, calculated as the additional (observed–expected) of SPM per 10,000 person-years at risk (PYR) among patients with index early-onset HNSCC. The observed and expected numbers of SPM and PYR in each subgroup stratified on the year of index HNSCC diagnosis, sex, age, race, and calendar year were obtained using SEER-9 data.

For objective 2, multivariate Poisson regression analyses were utilized to internally compare the risks of SPM according to the receipt of PORT (yes vs no) by estimating RRs and 95% confidence intervals (95% CIs) were then estimated, followed by the adjustment according to the age at index HNSCC diagnosis, stage of index HNSCC, latency (the period between HNSCC and SPM diagnosis) and sex. They were indirectly adjusted through calendar year and age by utilizing person-year as an exposure [27]. In addition, Fine-Gray’s competing risk models were used to estimate the cumulative incidence of SPM by time after the diagnosis of index HNSCC, and the cumulative risks of developing SPM after index HNSCC diagnosis [28].

Analyses were performed using SPSS version 22.0, Stata/MP 15.1, and R 3.5.1. Differences were considered statistically significant when the two-side P-value < 0.05.

## Results

### Patient characteristics and survival outcomes

**Fig. 1:**
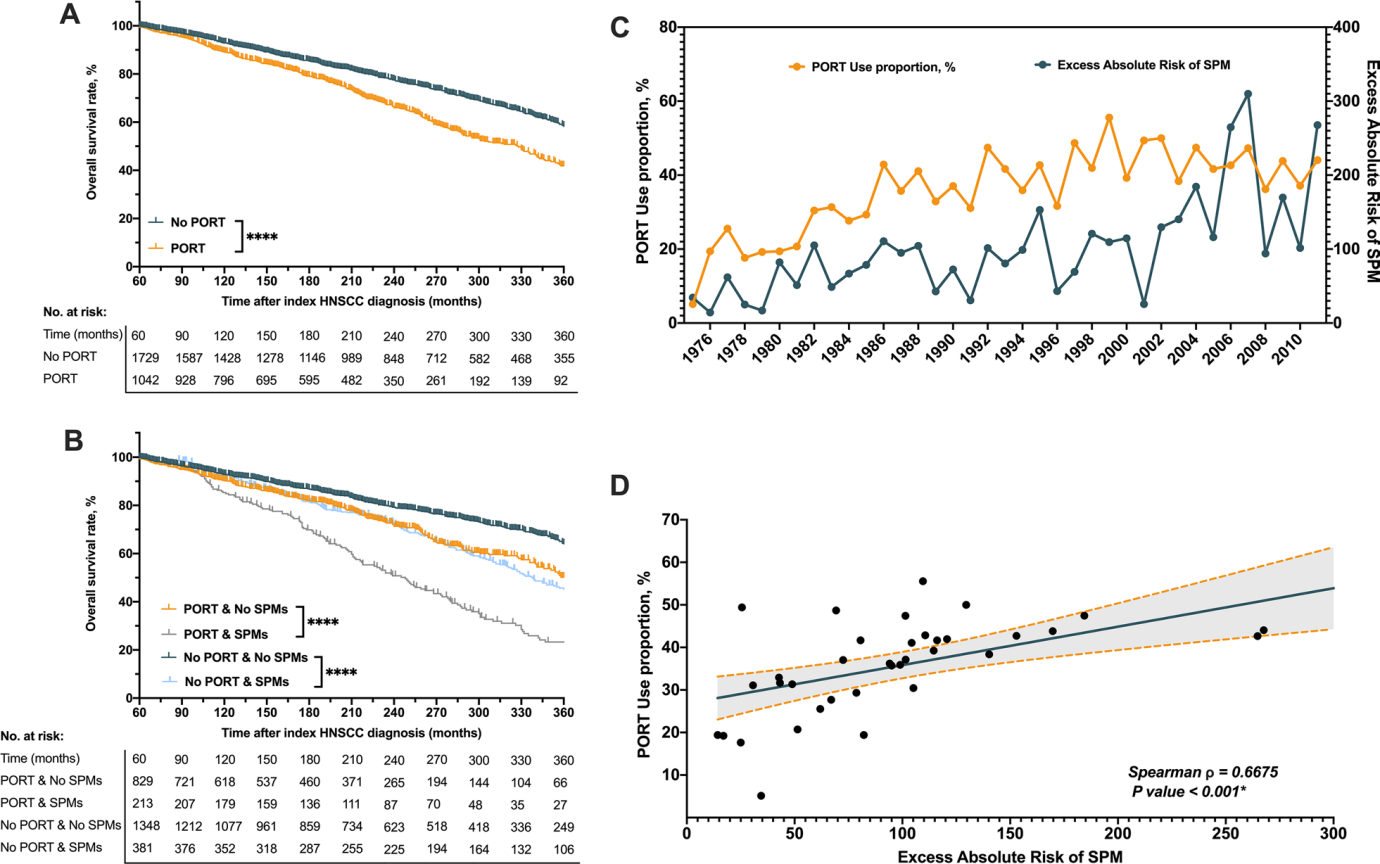
Kaplan–Meier curves of overall survival according to the receipt of PORT **A** and the combination of PORT receipt and SPM development **B** Trends in PORT use proportion and EAR among 5 year survivors of index young-onset HNSCC in the SEER-9 database **C** Spearman correlation between PORT use proportion and EAR **D** PORT, postoperative radiotherapy; SPM, second primary malignancy; EAR, excess absolute risk; HNSCC, head and neck squamous cell carcinoma

All 2771 5 year survivors diagnosed with young-onset, nonmetastatic HNSCC from 1975 to 2011 and who met the case criteria were enrolled in this study. Males (n = 2031, 73.3%) and whites (n = 2417, 87.2%) constituted most of the cases. Of the 2771 patients, 37.6% were treated with PORT. The receipt of PORT was the highest in black patients (57.9%), higher among patients who were diagnosed after the year 2000 (44.0%), and lower among patients younger than 35 years (28.5%). Unexpectedly, among the included patients who survived  ≥ 5 years, PORT-treated patients (10-year overall survival [OS], 92.9%) had worse long-term survival outcomes compared with non-PORT-treated patients (10 year OS, 89.1%, Fig. 1A, Additional file 1: Figure S1A).

Of the 2771 identified cases, 594 (21.4%) had developed SPM during the follow-up period (median, 173 months), with 213 (35.9%) among PORT-treated patients and 381 (64.1%) among non-PORT-treated patients. SPM was associated with worse survival outcomes among both the PORT-treated and non-PORT-treated cohorts (Fig. 1B, Additional file 1: Figure S1B). The additional information on the baseline characteristics of patients is shown in Table 1.

**Table 1:** Participant characteristics

Variables	Total^a^, no. (%)	PORT, .no (%)	No PORT, .no. (%)	P value
Total No. of events	2771	1042	1729	
Median follow-up time, months (IQR)	217(137, 311)	197 (125, 269)	235 (146, 339)	< 0.001
Age at diagnosis, year				< 0.001
0–35	641(23.1)	183(28.5)	458(71.5)	
> 35	2130(76.9)	859(40.3)	1271(59.7)	
Race				< 0.001
White	2417(87.2)	867(35.9)	1550(64.1)	
Black	197(7.1)	114(57.9)	83(42.1)	
Other^‡^	131(4.7)	57(43.5)	74(56.5)	
Unknown	26(0.9)	4(15.4)	22(84.6)	
Sex				0.772
Female	740(26.7)	275(37.2)	465(62.8)	
Male	2031(73.3)	767(37.8)	1264(62.2)	
Year of diagnosis				< 0.001
1975–2000	1871(67.5)	646(34.5)	1225(65.5)	
2001–2018	900(32.5)	396(44.0)	504(56.0)	
Site of index HNSCC				< 0.001
Oral cavity	1876(67.7)	420(22.4)	1456(77.6)	
Oropharynx	383(13.8)	323(84.3)	60(15.7)	
Larynx	490(17.7)	281(57.3)	209(42.7)	
Hypopharynx	22(0.8)	18(81.8)	4(18.2)	
Stage of index HNSCC				
Localized	1681(60.7)	320(19.0)	1361(81.0)	< 0.001
Regional	949(34.2)	669(70.5)	280(29.5)	
Unknown	141(5.1)	53(37.6)	88(62.4)	
Development of SPMs				0.322
No	2177(78.6)	829(38.1)	1348(61.9)	
Yes	594(21.4)	213(35.9)	381(64.1)	
Median follow-up time at event (any SPMs), months (IQR)	173(111, 253)	166(47.7)	182(52.3)	< 0.001

*PORT* postoperative radiotherapy, *HNSCC* head and neck squamous cell carcinoma, *SPMs* second primary malignancies, *IQR* interquartile range

^a^After exclusion based on the minimum latency span, the characteristics of 5 year survivors of index young-onset HNSCC were reported

^b^Other races in SEER database included Asian/Pacific Islander, American Indian and Alaska Native

### Risk of SPM in index HNSCC patients vs general population

The proportion in receipt of PORT over time was increased significantly from 5.1% in 1975 to 55.6% in 1999, parallel to the Average Annual Percent Change (AAPC) of 3.54 (95% CI 2.4–4.7; p < 0.05), and remained stable at 44.1% in 2011 (AAPC: − 0.99, 95% CI − 3.0–1.0; p = 0.297) (Fig. 1C). Among all the patients with index HNSCCs, the SIR of SPM was 2.12 (95% CI 1.95–2.29), corresponding to 83.45 excess SPMs per 10,000 PYRs. The EARs over time were steadily increased from 34.49 excess SPMs per 10,000 PYRs in 1975 to 267.62 in 2011, parallel to the AAPC of 4.71 (95% CI 3.3–6.1; p < 0.05) (Fig. 1D). Spearman’s rank correlation analysis showed a positive correlation between the proportion in receipt of PORT and EAR (Spearman ρ = 0.6675, 95% CI 0.43–0.82; p < 0.0001).

**Fig. 2:**
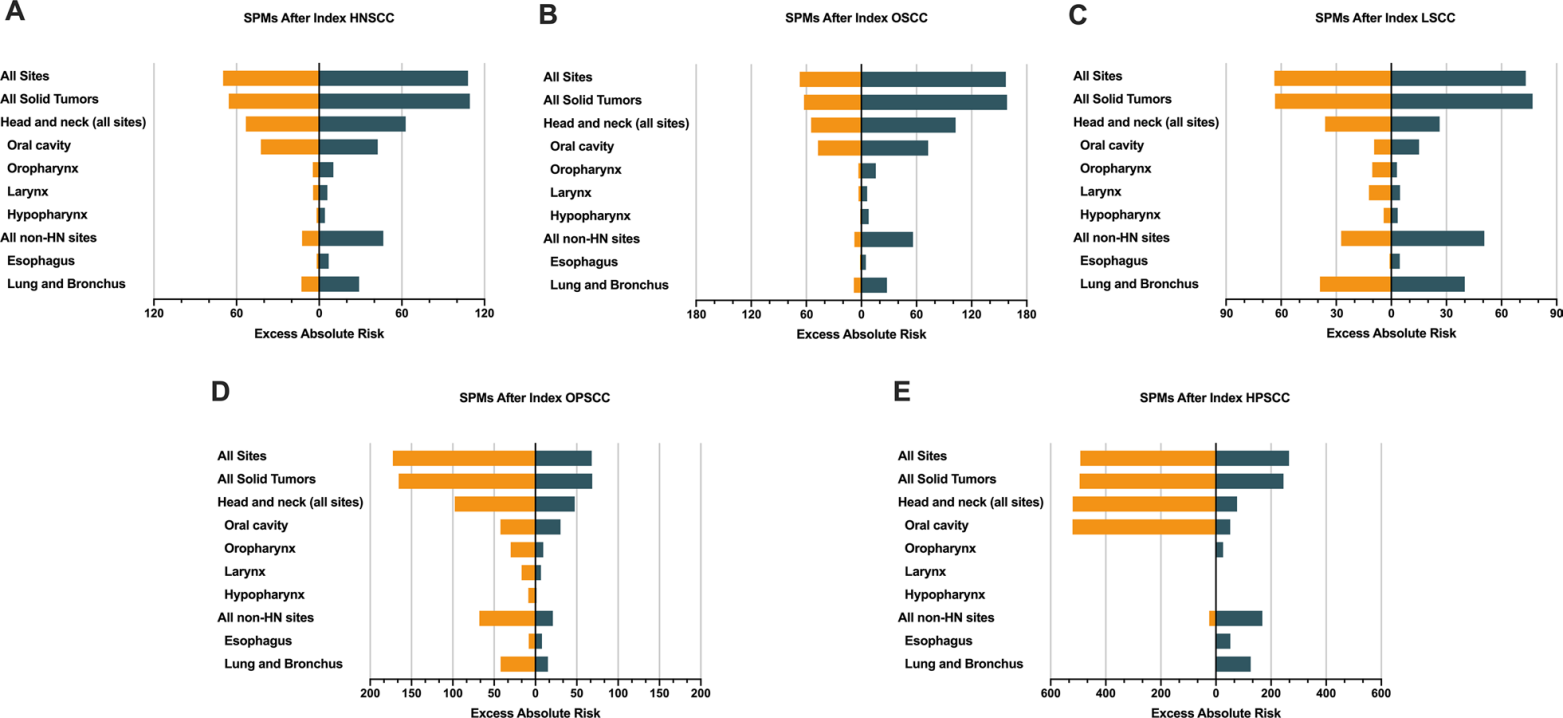
Excess absolute risks of second primary malignancy according to the receipt of postoperative radiotherapy, overall **A** and by the subsites of head and neck **B**–**E**

**Fig. 3:**
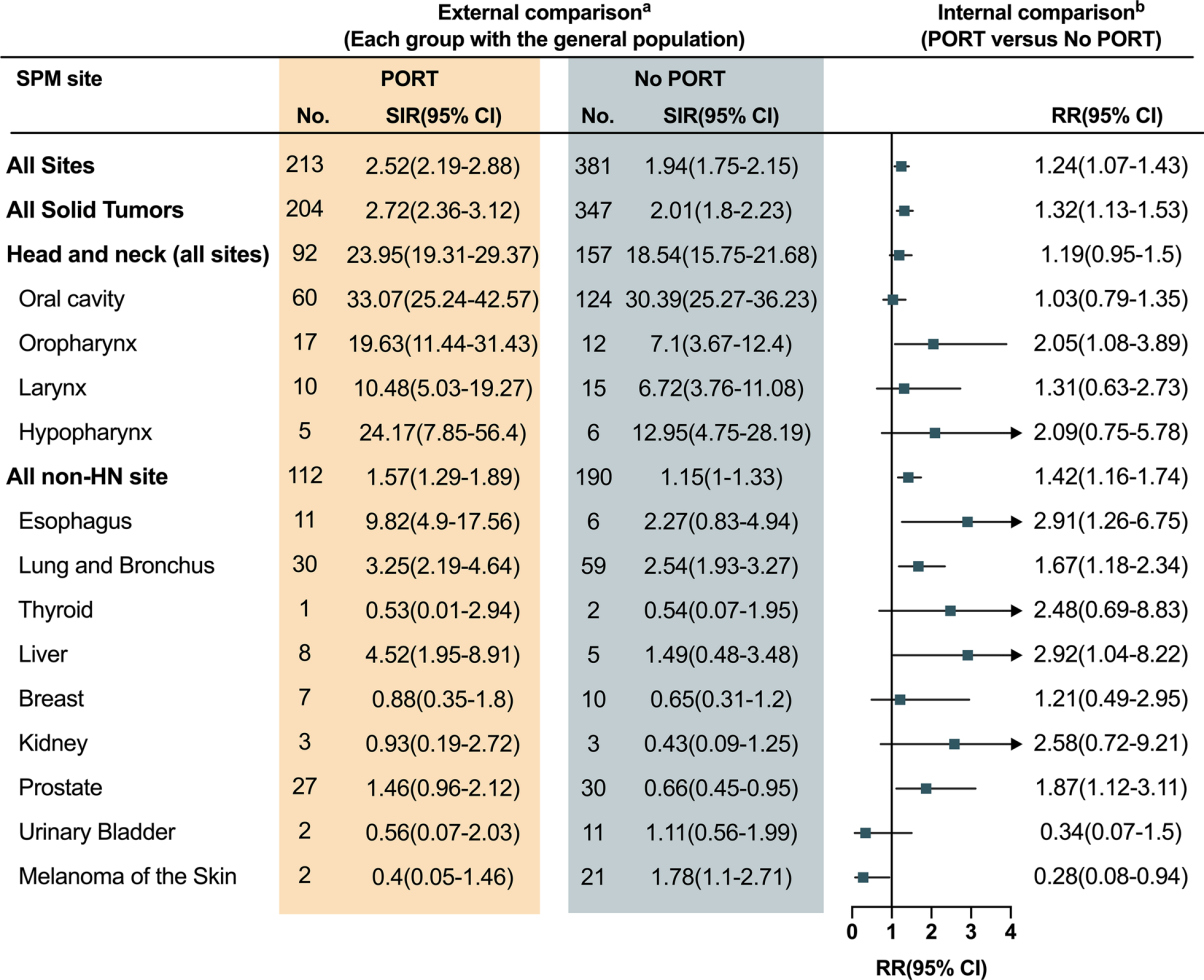
SIRs and RRs for second primary malignancy. **a** External comparison of PORT-associated risk by estimating SIRs, taking the general population as a reference. **b** Internal comparison of PORT-associated risk by estimating RRs. SIR, standardized incidence ratio; RR, relative risk; PORT, postoperative radiotherapy

When taking the general population as a reference, a higher risk of SPM was observed for PORT-treated HNSCC patients than that of non-PORT-treated patients, as measured by EARs (107.99 excess SPMs per 10,000 PYRs for PORT-treated patients and 69.73 for non-PORT-treated patients, Fig. 2A) and SIRs (2.52 [95% CI 2.19 to 2.88] for PORT-treated patients and 1.94 [95% CI 1.75 to 2.15] for non-PORT-treated patients, Fig. 3). Among PORT-treated HNSCC patients, the excess burden of SPM was the highest for head and neck cancers (62.84 excess SPMs per 10,000 PYRs), followed by lung and bronchus cancers (28.98 excess SPMs per 10,000 PYRs), and esophageal cancers (6.87 excess SPMs per 10,000 PYRs).

The excess burden of SPM and the association with PORT differed by the subsites of index head and neck cancer (Fig. 2B–E). Postoperative radiation was associated with a higher excess burden of SPM among index oral cavity SCC (EAR, 157.72) and laryngeal SCC (EAR, 73.21). In contrast, it was associated with a decreased excess burden of SPM among index oropharyngeal SCC (EAR, 67.97) and index hypopharyngeal SCC (EAR, 265.19). Specifically, compared with non-PORT-treated patients, the excess burden of SPM at most sites was higher for PORT-treated patients diagnosed with index oral cavity SCC (Fig. 2B) or laryngeal SCC (Fig. 2C). Conversely, it was relatively lower for PORT-treated patients diagnosed with oropharyngeal SCC (Fig. 2D) or hypopharyngeal SCC (Fig. 2E). In comparison, the excess burden of SPM at non-HN sites was higher for PORT-treated patients diagnosed with index hypopharyngeal SCC (Fig. 2E). Similar findings were also found by estimating the SIRs of SPM (Fig. 3, Additional file 1: Table S3).

### Risk of SPM in PORT-treated patients vs non-PORT-treated patients

**Fig. 4:**
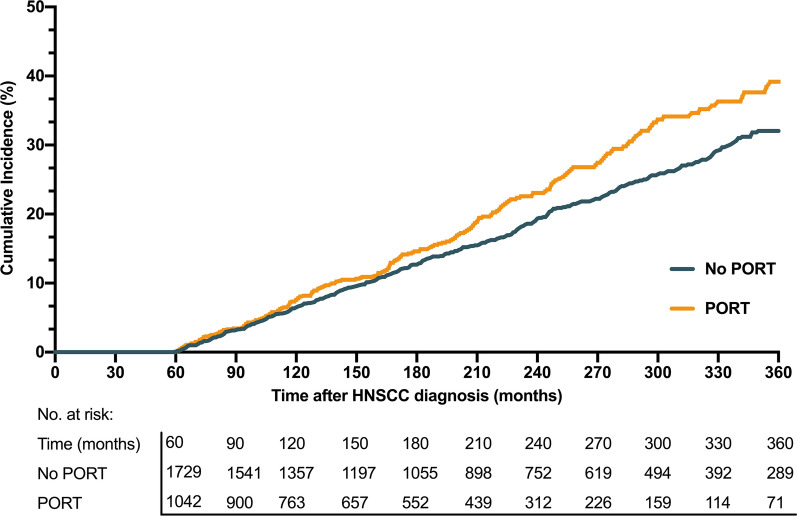
Cumulative incidence of SPM among 5 year survivors of index young-onset HNSCC, stratified by the receipt of PORT (yes vs no). SPM, second primary malignancy; PORT, postoperative radiotherapy; HNSCC, head and neck squamous cell carcinoma

The risk of SPM was also internally compared according to the receipt of PORT. The cumulative incidence of SPM at 15 years after the diagnosis of index HNSCC was 14.6% (95% CI 12.4–17.3%) for PORT-treated patients and 12.7% (95% CI 11.1–15.0%) for non-PORT-treated patients. The disparity was enhanced with the next follow-up (39.3% [95% CI 34.2–44.9%] for PORT-treated patients and 32.1% [95% CI 29.1–35.4%] for non-PORT-treated patients, at 30 years; Fig. 4).

The results of Poisson regression analyses revealed that PORT was associated with elevated risks of SPM (RR, 1.23; 95% CI 1.07–1.43; p = 0.004; Fig. 3). PORT-associated risk of SPM was elevated for the majority of sites, and was the highest for liver (RR, 2.92; 95% CI 1.04–8.22; p = 0.04), followed by esophagus (RR, 2.91; 95% CI 1.26–6.75; p = 0.01), oropharynx (RR, 2.05; 95% CI 1.08–3.89; p = 0.03), and lung and bronchus (RR, 1.67; 95% CI 1.18–2.34; p < 0.001).

The PORT-associated risks of SPM differed by the subsites of index head and neck cancer (Additional file 1 Table S3). PORT-associated risk of SPM was elevated among patients diagnosed with index oral cavity SCC (RR, 1.68; 95% CI 1.39–2.03) and laryngeal SCC (RR, 1.02; 95% CI 0.73–1.43). No significant changes in the risk were observed among PORT-treated patients diagnosed with oropharyngeal SCC (RR, 0.42; 95% CI 0.13–1.38) or hypopharyngeal SCC (RR, 0.7; 95% CI 0.14–3.37). Similar results were observed among patients younger than 40 years (Additional file 1Table S4).

**Fig. 5:**
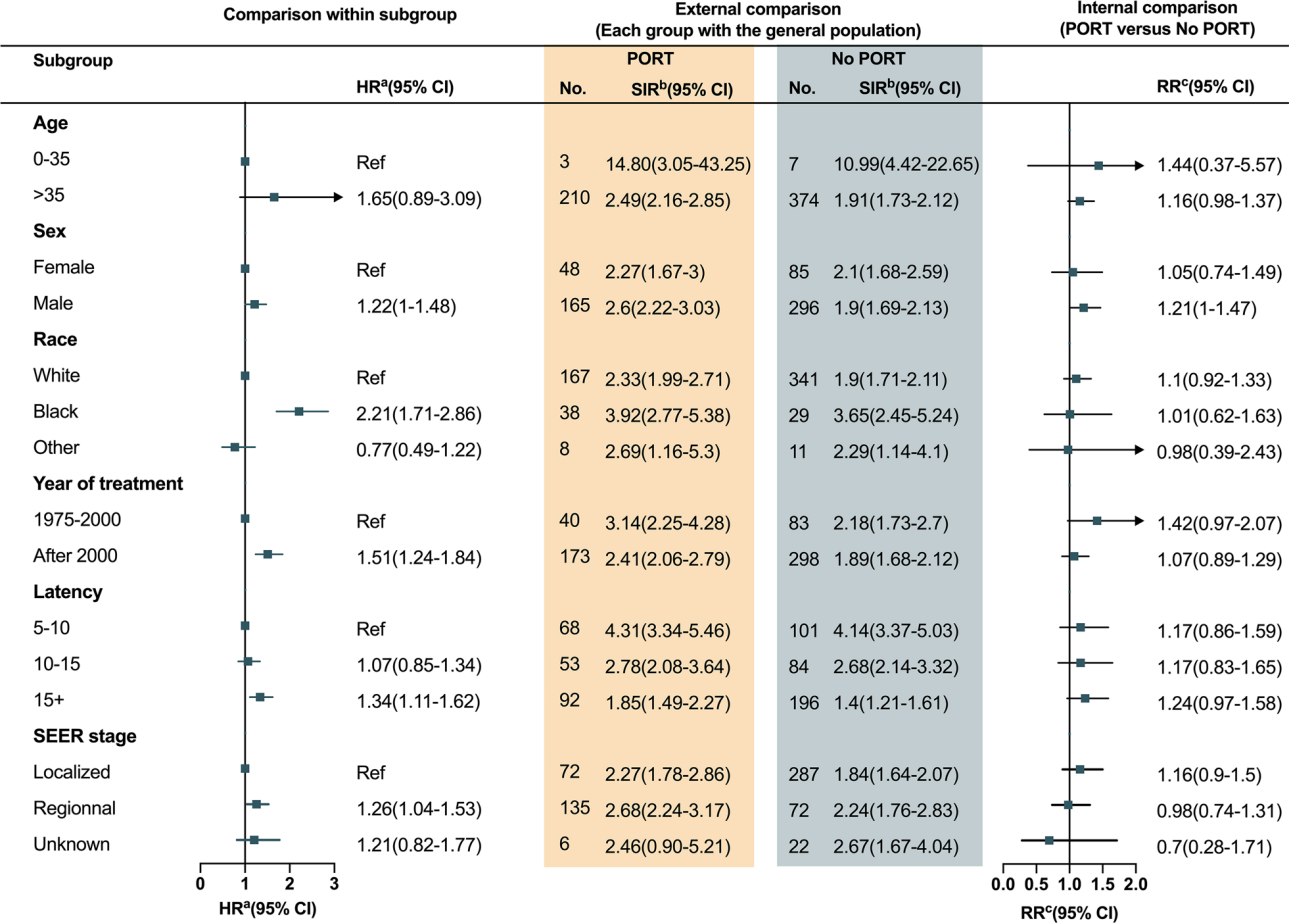
HRs, SIRs, and RRs for second primary malignancy, stratified by the demographic and clinical characteristics of patients. **a** Comparison of risk of SPM between subgroups by estimating HRs through Fine-Gray’s competing risk analyses. **b** External comparison of PORT-associated risk by estimating SIRs, taking the general population as a reference. **c** Internal comparison of PORT-associated risk by estimating RRs. *HR* hazard ratio, *SIR* standardized incidence ratio, *RR* relative risk, *PORT* postoperative radiotherapy

The results of Fine-Gray’s competing risk analysis (Fig. 5) showed a higher risk of developing SPM among index HNSCC patients who were aged > 35 years (Hazard ratio [HR], 1.65; 95% CI 0.89–3.09), male (HR 1.22; 95% CI 1–1.48), black race (HR 2.21; 95% CI 1.71–2.86), treated after the year 2000 (HR,1.51; 95% CI 1.24–1.84), or diagnosed with regional diseases (HR 1.26; 95% CI 1.04–1.53). The risk was increased with time since HNSCC diagnosis (HR 1.34; 95% CI 1.11–1.62). In addition, regarding PORT-associated risk, a relatively higher RR was found for patients younger than 35 years (RR, 1.44, 95% CI 0.37–5.57). The RRs were higher for those who were male (RR, 1.21, 95% CI 1–1.47), diagnosed before the year 2000 (RR 1.42, 95% CI 0.97–2.07), or diagnosed with localized diseases (RR 1.16, 95% CI 0.9–1.5). The RRs elevated for a more extended period since the diagnosis of index HNSCC, with a RR of 1.24 after 15 years (95% CI 0.97–1.58, Fig. 5).

## Discussion

PORT is an effective treatment to improve local control and prolong survival [10]. Nevertheless, adolescent and young patients are especially vulnerable to radiation-induced carcinogenesis and possess longer life expectancies to develop radiation-associated SPM [23]. This SEER-based study investigated the impact of PORT on developing SPM among adolescent and young patients who survived more than 5 years after the diagnosis of index HNSCC. We found a significantly positive correlation between the receipt of PORT and the long-term risk of developing SPM. Additionally, we conducted an internal comparison for the risk of developing SPM according to the receipt of PORT and further supported that the receipt of PORT was associated with an elevated risk of SPM, particularly exposure at a younger age, and the risk increased with longer follow-up. This study underscores the importance of long-term surveillance for these patients.

Evaluation of SIR and EAR, which was the highlight of most previous population-based studies on SPM [14, 29], could provide meaningful preliminary population-level data to support investigations on the risk of developing SPM relative to the United States general population. In this SEER-based study using cancer surveillance data from 1975 to 2011, we observed a relatively elevated excess risk of developing SPM among PORT-treated patients, with an EAR of 107.99 compared to that of 69.73 among non-PORT-treated patients. Accordingly, the highest risk ratio of SPM among PORT-treated patients was observed in the head and neck (SIR, 23.95), followed by esophagus (SIR, 9.82), liver (SIR, 4.52), and lung and bronchus (SIR, 3.25).

However, the disparities between early-onset HNSCC patients and the general population, including tobacco use, socioeconomic status, and health care access, may bias and confound the results of external comparisons [30–32]. Thus, the second objective of our investigations focused on the internal comparison, which was the greatest strength of this study compared with previous studies [14, 29]. Notably, since PORT was not randomly assigned in this retrospective study, the adverse cancer features that differed in PORT-treated vs non-PORT-treated patients (e.g. pT4 primary tumor, pN2 or pN3 nodal disease, and positive margin) were likely to confound the internal cohort comparisons. Nevertheless, the internal comparison was less likely to be confounded by the tobacco use and drinking history as these factors did not impact the receipt of PORT for index HNSCC [10]. Apart from the adverse cancer features, physician preference may be the major factor influencing the choice of PORT for HNSCC [1], and it is not likely to impact the risk of developing SPM.

Through internal cohort comparisons, elevated PORT-associated risks were observed for the cancers of head and neck (RR, 1.19), esophagus (RR, 2.91), lung and bronchus (RR, 1.67), and thyroid (RR, 2.48), which were closed to irradiation fields and were more likely to be exposed to radiation leakage [33]. The development of techniques from three-dimensional conformal radiotherapy (3D-CRT) to intensity-modulated radiotherapy (IMRT) has allowed more accurate dose distributions, thus minimizing treatment toxicity and improving the survival rates [34]. However, more irradiation fields and monitor units were required for IMRT to modulate fluency, leading to longer irradiation times and increased dose for collimator scatter and head leakage [33]. The impact of IMRT on the risk of developing SPM requires further investigation. Previous studies of other cohorts exposed to radiation have reported a significant dose–response between the exposure of radiation and SPM [35, 36]. This study showed a reduced RR among patients diagnosed after 2000, indicating shorter follow-up periods and lower levels of radiation leakage from the improved radiation techniques.

The overall radiation-related risks of developing SPM differed by index HNSCC subsites, which were elevated among cases with index oral cavity SCC or laryngeal SCC, but not significantly decreased among patients with index oropharyngeal SCC and hypopharyngeal SCC. Specifically, for the patients with index oral cavity SCC, the highest burden of SPM was recognized in the region of head and neck (SIR, 48.34; RR, 1.88). For patients diagnosed with index laryngeal SCC or hypopharyngeal SCC, strongly elevated burdens of SPM were observed in esophagus (LSCC [SIR, 4.49; RR, 1965.58]; HPSCC [SIR, 55.6; RR, 144.88]), lung and bronchus (LSCC [SIR, 5.01; RR, 1]; HPSCC [SIR, 18.73; RR, 458.03]). These differences in the index HNSCC subsites were consistent with previous studies [14, 37, 38]. The variations observed may be caused by the exposure of adjacent tissues to a higher dose of radiation leakage.

Compared with other races, the black race was related to a higher risk of developing SPM (HR, 2.21), which was in agreement with previous studies [39]. However, black patients with index young-onset HNSCC were less likely to suffer from radiation-induced SPM, which required confirmation by further large-scale clinical observation. Regarding sex, male patients were more prone to developing SPM than female patients due to a higher prevalence of tobacco smoking among male patients, similar to previous reports [31]. A higher radiation-related risk of SPM was also observed among male patients, warranting further confirmations and investigations.

RRs and HRs stratified by the follow-up times and the cumulative incidence curve consistently showed increased long-term risks (elevating with time) for radiation-associated SPM, which were in agreement with previous studies [35, 36]. A higher radiation-associated risk of developing SPM was observed among index HNSCC patients with younger age (age ≤ 35 years; RR 1.44) and with localized diseases (RR, 1.16), as these patients had a longer life expectancy to be affected by the radiation exposure. This finding was consistent with our result that the radiation-associated risk of SPM was increased with time.

Multiple studies have reported the negative influence of SPM on the survival of patients with index HNSCC [40–42]. Indeed, our study shows that the development of SPM leads to a lower survival outcome in both the PORT- and non-PORT-treated patients. Furthermore, we reveal that PORT increases the long-term risk of SPM. The findings of our study can help improve the follow-up surveillance strategies, which will benefit the adolescent and young patients with HNSCC, especially those with oral cavity SCC or laryngeal SCC. Notably, these findings should be interpreted cautiously, and further investigations and assessments of PORT in managing young-onset HNSCC are warranted to verify our results.

Several important limitations of this study should be considered. First, similar to most databases, the SEER had intrinsic selection bias and unmeasured confounding. Since PORT is administrable in outpatient clinics, PORT would likely be under ascertained if not documented, which may skew the estimations of RR toward the null. Furthermore, lack of the detailed data on postoperative radiation (e.g. the dose of radiation) has limited our investigation of the dose–response relationship between PORT and the development of SPM, although PORT is performed under the guidance of cancer treatment guidelines in most cases, which have recommended standard dose and volume. Future prospective studies should investigate the risk adapted de-intensification of postoperative radiotherapy for young patients in an effort to maximize survival benefits. Information on family history of cancer, smoking history, alcohol consumption, and health care access are also lacking, which could provide additional insights. Future studies are warranted to evaluate the correlation between these factors and PORT-associated risks of SPM development. Finally, previous studies into field tumorigenesis have reported abnormalities (e.g. loss of heterozygosity, high Ki-67 proliferation index, and TP53 gene mutation) in histologically normal tissues adjacent to the cancer of head and neck [43]. It is possibly that cancer recurred within the area of preexisting genetic field tumorigenesis. Thus, our study focused on patients who survived longer than 5 years after diagnosing with index young-onset HNSCC. Despite these limitations, this is the first population-based study to assess the PORT-related SPM risk among 5 year survivors of index young-onset HNSCC. The methodological strengths of our study, including the large size of samples, near-complete follow-up duration, and the internal comparisons, maximize the generalizability and validity of results.

Taken together, this study shows that the development of SPM leads to a lower survival outcome in adolescent and young patients with index HNSCC. Furthermore, PORT is revealed to increase the long-term risk of SPM, based on our study of 5 year survivors with index young-onset HNSCC.

## Supplementary Information

**Additional file 1: Table S1.** Definition of index squamous cell carcinoma of head and neck used in the present study. **Table S2.** Definition of second primary malignancy used in the present study. **Table S3.** Observed number, standardized incidence ratios (SIRs), and PORT-associated relative risks (RRs) for overall and specific SPMs, stratified by the receipt of PORT and the subsites of head and neck among 5-year survivors of patients diagnosed with index young-onset HNSCC. **Table S4.** Observed number, standardized incidence ratios (SIRs), and PORT-associated relative risks (RRs) for overall and specific SPMs, stratified by the receipt of PORT among 5-year survivors of young-onset HNSCC before 40 years of age. **Figure S1**. Kaplan-Meier curves of cancer-specific survival according to the receipt of PORT (**A**) and the combination of PORT receipt and SPM development (**B**). **Table S1**. Definition of index squamous cell carcinoma of head and neck used in the present study. **Table S2**. Definition of second primary malignancy used in the present study. **Table S3.** Observed number, standardized incidence ratios (SIRs), and PORT-associated relative risks (RRs) for overall and specific SPMs, stratified by the receipt of PORT and the subsites of head and neck among 5-year survivors of patients diagnosed with index young-onset HNSCC. **Table S4.** Observed number, standardized incidence ratios (SIRs), and PORT-associated relative risks (RRs) for overall and specific SPMs, stratified by the receipt of PORT among 5-year survivors of young-onset HNSCC before 40 years of age.

## Data Availability

The data analyzed in this study is available at https://seer.Cancer.gov/.

## Abbreviations

SPM: Second primary malignancy
HNSCC: Head and neck squamous cell carcinoma
SCC: Squamous cell carcinoma
PORT: Postoperative therapy
SIR: Standardized incidence ratio
EAR: Excess absolute risk
RR: Relative risk
SEER: The surveillance, epidemiology, and end results
AJCC: The american joint committee on cancer
PYR: Person-years at risk
CI: Confidence interval
OS: Overall survival
AAPC: The average annual percent change
IMRT: Intensity-modulated radiotherapy
3D-CRT: Three-dimensional conformal radiotherapy
