# Supplementary material for: Association between postoperative radiotherapy for young-onset head and neck cancer and long-term risk of second primary malignancy: a population-based study
Source: J Transl Med. 2022 Sep 5;20:405. doi: 10.1186/s12967-022-03544-y (PMC9446763; doi:10.1186/s12967-022-03544-y)

**Additional file**

**CONTENTS**

**Additional file Tables**

**Additional file 1 Table S1**. Definition of index squamous cell carcinoma of head and neck used in the present study

**Additional file 1 Table S2**. Definition of second primary malignancy used in the present study

**Additional file 1 Table S3.** Observed number, standardized incidence ratios (SIRs), and PORT-associated relative risks (RRs) for overall and specific SPMs, stratified by the receipt of PORT and the subsites of head and neck among 5-year survivors of patients diagnosed with index young-onset HNSCC

**Additional file 1 Table S4.** Observed number, standardized incidence ratios (SIRs), and PORT-associated relative risks (RRs) for overall and specific SPMs, stratified by the receipt of PORT among 5-year survivors of young-onset HNSCC before 40 years of age

**Additional Figures**

**Additional file1 Figure S1**. Kaplan-Meier curves of cancer-specific survival according to the receipt of PORT (A) and the combination of PORT receipt and SPM development (B).

| **Additional file 1 Table S1**. Definition of index squamous cell carcinoma of head and neck used in the present study | | | | |
| --- | --- | --- | --- | --- |
|  | **ICD-O-3 Histology (Type)** | **Subsite** | **ICD-O-3 Site** | **SITE** |
| **Head and neck** | 8070/3 8071/3 8072/3 8073/3 8074/3 8075/3 8076/3 8077/3 8078/3 | Oral cavity | C000-C006,C008-C009 | Lip |
|  |  |  | C020-C023,C028-C029 | Tongue (Excluding base of tongue and lingual tonsil) |
|  |  |  | C030-C031,C039-C041,C048-C050,C058-C062,C068-C069 | Gum, floor of mouth, & other mouth (Excluding soft palate and uvula) |
|  |  | Oropharynx | C019 | Base of tongue, NOS |
|  |  |  | C024 | Lingual tonsil |
|  |  |  | C051 | Soft palate, NOS |
|  |  |  | C052 | Uvula |
|  |  |  | C090-C091,C098-C104,C108-C109 | Oropharynx |
|  |  | Larynx | C320-C323,C328-C329 | Larynx |
|  |  | Hypopharynx | C129-C132,C138-C139 | Hypopharynx |
|  |  | Other and ill-defined sites in the lip, oral cavity and pharynx | C140, C142, C148 | Other and ill-defined sites in the lip, oral cavity and pharynx |

| **Additional file 1 Table S2**. Definition of second primary malignancy used in the present study | |
| --- | --- |
|  | **ICD-O-3 Site** |
| **All Sites** | All sites |
| **All Solid Tumors** | All excluding C420, C421, C424 |
| **Head and neck (all sites)** | C000-C006,C008-C009, C019-C024,C028-C031,C039-C041,C048-C052,C058-C062,C068-C069, C090-C091,C098-C104,C108-C109 |
| Oral cavity | C000-C006,C008-C009 |
|  | C020-C023,C028-C029 |
|  | C030-C031,C039-C041,C048-C050,C058-C062,C068-C069 |
| Oropharynx | C019 |
|  | C024 |
|  | C051 |
|  | C052 |
|  | C090-C091,C098-C104,C108-C109 |
| Larynx | C320-C323,C328-C329 |
| Hypopharynx | C129-C132,C138-C139 |
| Other and ill-defined sites in the lip, oral cavity and pharynx | C140, C142, C148 |
| **All non-HN site** | All excluding C000-C006,C008-C009, C019-C024,C028-C031,C039-C041,C048-C052,C058-C062,C068-C069, C090-C091,C098-C104,C108-C109, C420, C421, C424 |
| Esophagus | C150-C155,C158-C159 |
| Lung and Bronchus | C340-C343,C348-C349 |
| Thyroid | C739 |
| Liver | C220 |
| Breast | C500-C506,C508-C509 |
| Kidney | C649 |
| Prostate | C619 |
| Urinary Bladder | C670-C679 |
| Melanoma of the Skin | C440-C449 |

| **Additional file 1 Table S3.** Observed number, standardized incidence ratios (SIRs), and PORT-associated relative risks (RRs) for overall and specific SPMs, stratified by the receipt of PORT and the subsites of head and neck among 5-year survivors of patients diagnosed with index young-onset HNSCC | | | | | |
| --- | --- | --- | --- | --- | --- |
|  | **External comparison (Each group with the general population)** | | | | **Internal comparison (PORT versus No PORT)** |
| **SPM site** | **PORT** |  | **No PORT** |  |  |
|  | **No.** | **SIR(95%CI)** | **No.** | **SIR(95%CI)** | **RR(95% CI)** |
| **OSCC** |  |  |  |  |  |
| **All Sites** | 103 | **3.68(3-4.46)** | 314 | **1.95(1.74-2.18)** | **1.68(1.39-2.03)** |
| **All Solid Tumors** | 100 | **4.02(3.27-4.89)** | 285 | **2.00(1.78-2.25)** | **1.81(1.49-2.2)** |
| **Head and neck (all sites)** | 58 | **48.34(36.7-62.49)** | 137 | **19.72(16.55-23.31)** | **1.88(1.44-2.47)** |
| Oral cavity | 41 | **71.29(51.16-96.72)** | 116 | **34.55(28.55-41.44)** | **1.56(1.14-2.14)** |
| Oropharynx | 10 | **37.00(17.74-68.05)** | 8 | **5.69(2.46-11.21)** | **4.44(1.95-10.12)** |
| Larynx | 4 | **13.73(3.74-35.16)** | 9 | **4.97(2.27-9.43)** | 1.96(0.68-5.68) |
| Hypopharynx | 3 | **47.41(9.78-138.54)** | 4 | **10.71(2.92-27.42)** | **6.14(1.71-21.98)** |
| **All non-HN site** | 42 | **1.77(1.28-2.4)** | 148 | 1.09(0.93-1.29) | **1.73(1.31-2.29)** |
| Esophagus | 3 | **8.84(1.82-25.85)** | 4 | 1.85(0.5-4.73) | 2.25(0.65-7.78) |
| Lung and Bronchus | 6 | 2.06(0.76-4.49) | 38 | **2.01(1.43-2.77)** | **2.19(1.32-3.62)** |
| Thyroid | 0 | 0(0-4.87) | 2 | 0.63(0.08-2.29) | 3.2(0.64-16.01) |
| Liver | 3 | **5.2(1.07-15.21)** | 5 | 1.78(0.58-4.16) | 2.31(0.57-9.31) |
| Breast | 4 | 1.21(0.33-3.11) | 7 | 0.56(0.22-1.15) | 1.74(0.52-5.79) |
| Kidney | 1 | 0.96(0.02-5.32) | 3 | 0.52(0.11-1.51) | 4.02(0.8-20.23) |
| Prostate | 10 | 1.86(0.89-3.41) | 23 | 0.62(0.39-0.93) | **2.12(1.01-4.44)** |
| Urinary Bladder | 1 | 0.91(0.02-5.06) | 9 | 1.11(0.51-2.1) | 0.48(0.06-3.79) |
| Melanoma of the Skin | 2 | 1.19(0.14-4.32) | 20 | **2.03(1.24-3.13)** | 0.61(0.18-2.04) |
| **LSCC** |  |  |  |  |  |
| **All Sites** | 61 | **1.93(1.48-2.48)** | 51 | **1.67(1.25-2.2)** | 1.02(0.73-1.43) |
| **All Solid Tumors** | 59 | **2.1(1.6-2.71)** | 47 | **1.74(1.28-2.32)** | 1.06(0.75-1.5) |
| **Head and neck (all sites)** | 15 | **10.51(5.88-17.34)** | 15 | **11.33(6.34-18.69)** | 0.77(0.39-1.51) |
| Oral cavity | 8 | **12.07(5.21-23.79)** | 5 | **7.95(2.58-18.55)** | 1.57(0.53-4.7) |
| Oropharynx | 2 | 6.89(0.83-24.88) | 3 | **12.53(2.58-36.62)** | 0.33(0.06-1.69) |
| Larynx | 3 | **7.71(1.59-22.54)** | 5 | **13.32(4.32-31.08)** | 0.44(0.11-1.75) |
| Hypopharynx | 2 | **23.57(2.85-85.15)** | 2 | **25.13(3.04-90.78)** | 0.89(0.12-6.28) |
| **All non-HN site** | 44 | **1.65(1.2-2.22)** | 32 | **1.25(0.85-1.76)** | 1.19(0.8-1.79) |
| Esophagus | 2 | 4.49(0.54-16.22) | 0 | 0(0-8.59) | 1965.58(5.21E-22-7.42E+27) |
| Lung and Bronchus | 19 | **5.01(3.01-7.82)** | 17 | **4.33(2.52-6.93)** | 1(0.56-1.76) |
| Thyroid | 1 | 1.77(0.04-9.84) | 0 | 0(0-8.09) | 1.58(0.14-17.47) |
| Liver | 1 | 1.65(0.04-9.19) | 0 | 0(0-7.77) | 739.33(1.91E-22-2.85E+27) |
| Breast | 2 | 0.79(0.1-2.85) | 3 | 1.36(0.28-3.97) | 0.55(0.09-3.29) |
| Kidney | 0 | 0(0-3.21) | 0 | 0(0-3.54) | 0(2.05E-28-3.06E+21) |
| Prostate | 11 | 1.43(0.71-2.56) | 5 | 0.68(0.22-1.58) | 1.95(0.68-5.6) |
| Urinary Bladder | 1 | 0.7(0.02-3.91) | 2 | 1.25(0.15-4.51) | 0.44(0.04-4.88) |
| Melanoma of the Skin | 0 | 0(0-2.18) | 0 | 0(0-2.17) | 0.89(1.06E-35-7.45E+34) |
| **OPSCC** |  |  |  |  |  |
| **All Sites** | 44 | **1.88(1.37-2.53)** | 15 | **3.42(1.91-5.64)** | 0.42(0.13-1.38) |
| **All Solid Tumors** | 41 | **1.99(1.43-2.7)** | 14 | **3.59(1.96-6.02)** | 0.43(0.13-1.39) |
| **Head and neck (all sites)** | 19 | **16.98(10.22-26.52)** | 4 | **20.5(5.59-52.49)** | 0.62(0.27-1.44) |
| Oral cavity | 11 | **20.53(10.25-36.73)** | 2 | **21.34(2.58-77.1)** | 0.9(0.26-3.12) |
| Oropharynx | 5 | **17.55(5.7-40.96)** | 1 | **22.36(0.57-124.6)** | 0.45(0.09-2.32) |
| Larynx | 3 | **12.17(2.51-35.56)** | 1 | 21.43(0.54-119.4) | 0.75(0.08-6.74) |
| Hypopharynx | 0 | 0(0-71.59) | 0 | 0(0-367.22) | 0.0002141(5.54E-29-8.27E+20) |
| **All non-HN site** | 22 | 1.13(0.71-1.71) | 10 | **2.7(1.29-4.96)** | 0.48(0.25-0.92) |
| Esophagus | 5 | **16.28(5.29-38)** | 2 | **37.47(4.54-135.34)** | 0.47(0.09-2.47) |
| Lung and Bronchus | 2 | **0.87(0.1-3.13)** | 4 | **8.36(2.28-21.4)** | 0.40(0.11-1.36) |
| Thyroid | 0 | 0(0-6.69) | 0 | 0(0-35.23) | 0.21(1.76E-38-2.5E+36) |
| Liver | 4 | **7.48(2.04-19.14)** | 0 | 0(0-47.36) | 787.97(2.11E-22-2.95E+27) |
| Breast | 1 | 0.48(0.01-2.66) | 0 | 0(0-6.51) | 0.65(0.06-7.15) |
| Kidney | 2 | 2.09(0.25-7.55) | 0 | 0(0-23.02) | 419.03(1.11E-22-1.58E+27) |
| Prostate | 6 | 1.2(0.44-2.62) | 2 | 2.43(0.29-8.77) | 0.57(0.12-2.76) |
| Urinary Bladder | 0 | 0(0-3.75) | 0 | 0(0-21.58) | 0.21(1.76E-38-2.5E+36) |
| Melanoma of the Skin | 0 | 0(0-2.41) | 1 | 3.8(0.1-21.16) | 0.0001907(4.9E-29-7.42E+20) |
| **HPSCC** |  |  |  |  |  |
| **All Sites** | 5 | **5.88(3.04-10.27)** | 1 | **17.94(2.17-64.81)** | 0.7(0.14-3.37) |
| **All Solid Tumors** | 4 | **6.09(3.04-10.89)** | 1 | **20.17(2.44-72.85)** | 0.62(0.13-3) |
| **Head and neck (all sites)** | 0 | **24.48(5.05-71.53)** | 1 | **401.87(48.67-1451.7)** | 0.19(0.03-1.26) |
| Oral cavity | 0 | **37.51(4.54-135.49)** | 1 | **916.47(110.99-3310.62)** | 0.13(0.01-1.12) |
| Oropharynx | 0 | 38.93(0.99-216.9) | 0 | 0(0-3879.24) | 99.71(2.58E-23-3.85E+26) |
| Larynx | 0 | 0(0-107.55) | 0 | 0(0-2598.66) | 0.12(3.23E-37-4.18E+34) |
| Hypopharynx | 0 | 0(0-398.21) | 0 | 0(0-8700.59) | 0.12(3.23E-37-4.18E+34) |
| **All non-HN site** | 4 | **4.75(2.05-9.36)** | 0 | 0(0-39.16) | 747.11(1.99E-22-2.81E+27) |
| Esophagus | 1 | **55.6(6.73-200.83)** | 0 | 0(0-2793.6) | 144.88(3.78E-23-5.56E+26) |
| Lung and Bronchus | 3 | **18.73(6.08-43.71)** | 0 | 0(0-316.33) | 458.03(1.21E-22-1.73E+27) |
| Thyroid | 0 | 33.92(0.86-189.01) | 0 | 0(0-1337.22) | 128.79(3.33E-23-4.97E+26) |
| Liver | 0 | 0(0-65.42) | 0 | 0(0-1627.87) | 0.12(3.23E-37-4.18E+34) |
| Breast | 0 | 0(0-31.06) | 0 | 0(0-152.65) | 0.12(3.23E-37-4.18E+34) |
| Kidney | 0 | 0(0-45.45) | 0 | 0(0-1027.44) | 0.12(3.23E-37-4.18E+34) |
| Prostate | 0 | 0(0-7.59) | 0 | 0(0-289.29) | 0.12(3.23E-37-4.18E+34) |
| Urinary Bladder | 0 | 0(0-55.3) | 0 | 0(0-1967.82) | 0.12(3.23E-37-4.18E+34) |
| Melanoma of the Skin | 0 | 0(0-49.02) | 0 | 0(0-1242.79) | 0.12(3.23E-37-4.18E+34) |

| **Additional file 1 Table S4.** Observed number, standardized incidence ratios (SIRs), and PORT-associated relative risks (RRs) for overall and specific SPMs, stratified by the receipt of PORT among 5-year survivors of young-onset HNSCC before 40 years of age | | | | | |
| --- | --- | --- | --- | --- | --- |
|  | **External comparison (Each group with the general population)** | | | | **Internal comparison (PORT versus No PORT)** |
| **SPM site** | **PORT** | | **No PORT** | |  |
|  | **No.** | **SIR(95%CI)** | **No.** | **SIR(95%CI)** | **RR(95% CI)** |
| **All Sites** | 76 | **2.51(1.97-3.14)** | 179 | **2.12(1.82-2.46)** | 1.09(0.83-1.43) |
| **All Solid Tumors** | 73 | **2.71(2.13-3.41)** | 161 | **2.17(1.84-2.53)** | 1.16(0.88-1.53) |
| **Head and neck (all sites)** | 39 | **30.71(21.84-41.99)** | 86 | **23.54(18.83-29.08)** | 1.14(0.78-1.66) |
| Oral cavity | 28 | **45.58(30.29-65.87)** | 73 | **40.8(31.98-51.3)** | 0.94(0.61-1.46) |
| Oropharynx | 3 | **10.14(2.09-29.65)** | 3 | 3.74(0.77-10.92) | 2.74(0.55-13.67) |
| Larynx | 6 | **20.31(7.45-44.21)** | 7 | **7.94(3.19-16.35)** | 2.42(0.81-7.25) |
| Hypopharynx | 2 | **31.09(3.77-112.32)** | 3 | **16.74(3.45-48.91)** | 1.62(0.27-9.83) |
| **All non-HN site** | 34 | 1.33(0.92-1.85) | 75 | 1.06(0.83-1.33) | 1.19(0.79-1.79) |
| Esophagus | 4 | **11.46(3.12-29.35)** | 0 | 0(0-3.46) | 106456.4(0-1.4E+100) |
| Lung and Bronchus | 6 | 2.03(0.75-4.43) | 23 | **2.64(1.67-3.96)** | 0.71(0.29-1.75) |
| Thyroid | 0 | 0(0-3.94) | 1 | 0.47(0.01-2.64) | 0(0-3.71E+91) |
| Liver | 2 | 3.51(0.42-12.67) | 4 | 2.68(0.73-6.86) | 1.43(0.26-7.88) |
| Breast | 4 | 0.96(0.26-2.47) | 3 | 0.37(0.08-1.09) | 2.5(0.56-11.19) |
| Kidney | 0 | 0(0-3.26) | 0 | 0(0-1.17) | 2.71(0-7.7E+136) |
| Prostate | 6 | 1.15(0.42-2.5) | 11 | 0.63(0.31-1.12) | 1.67(0.62-4.51) |
| Urinary Bladder | 1 | 0.91(0.02-5.08) | 4 | 1.05(0.29-2.68) | 0.72(0.08-6.5) |
| Melanoma of the Skin | 1 | 0.51(0.01-2.86) | 12 | 2.09(1.08-3.65) | 0.21(0.03-1.64) |

**Figure S1**. Kaplan-Meier curves of cancer-specific survival according to the receipt of PORT (A) and the combination of PORT receipt and SPM development (B).


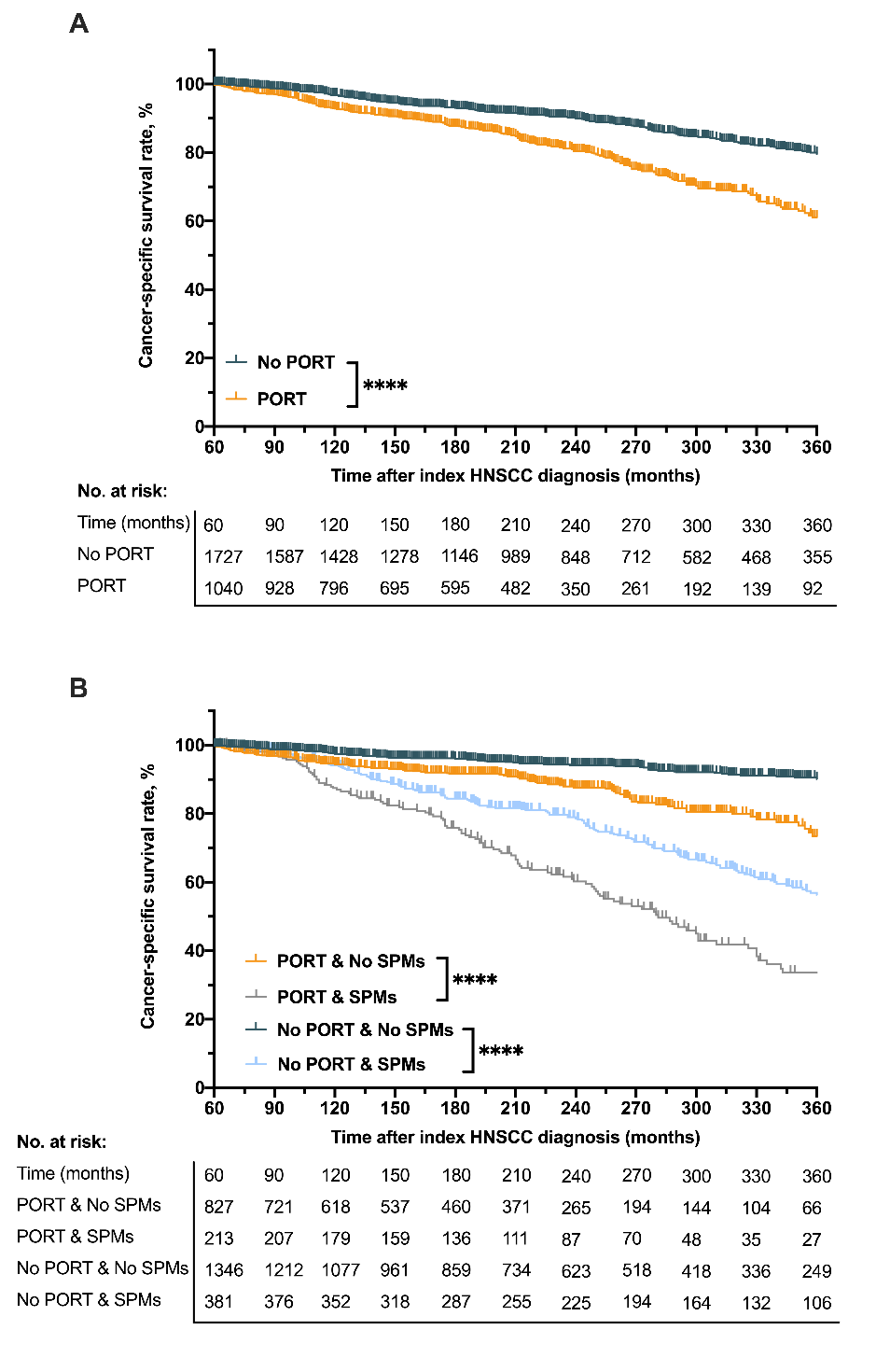

Supplement: Supplementary file 1 — Additional file 1: Table S1. Definition of index squamous cell carcinoma of head and neck used in the present study. Table S2. Definition of second primary malignancy used in the present study. Table S3. Observed number, standardized incidence ratios (SIRs), and PORT-associated relative risks (RRs) for overall and specific SPMs, stratified by the receipt of PORT and the subsites of head and neck among 5-year survivors of patients diagnosed with index young-onset HNSCC. Table S4. Observed number, standardized incidence ratios (SIRs), and PORT-associated relative risks (RRs) for overall and specific SPMs, stratified by the receipt of PORT among 5-year survivors of young-onset HNSCC before 40 years of age. Figure S1. Kaplan-Meier curves of cancer-specific survival according to the receipt of PORT (A) and the combination of PORT receipt and SPM development (B). Table S1. Definition of index squamous cell carcinoma of head and neck used in the present study. Table S2. Definition of second primary malignancy used in the present study. Table S3. Observed number, standardized incidence ratios (SIRs), and PORT-associated relative risks (RRs) for overall and specific SPMs, stratified by the receipt of PORT and the subsites of head and neck among 5-year survivors of patients diagnosed with index young-onset HNSCC. Table S4. Observed number, standardized incidence ratios (SIRs), and PORT-associated relative risks (RRs) for overall and specific SPMs, stratified by the receipt of PORT among 5-year survivors of young-onset HNSCC before 40 years of age. [file 12967_2022_3544_MOESM1_ESM.docx]
